# Association of Influenza Vaccination and Reduced Risk of Stroke Hospitalization among the Elderly: A Population-Based Case-Control Study

**DOI:** 10.3390/ijerph110403639

**Published:** 2014-04-02

**Authors:** Hui-Chen Lin, Hui-Fen Chiu, Shu-Chen Ho, Chun-Yuh Yang

**Affiliations:** 1 Department of Public Health, College of Health Sciences, Kaohsiung Medical University, Kaohsiung 807, Taiwan; E-Mails: lhuichen@cdc.gov.tw (H.-C.L.); u9391006@kmu.edu.tw (S.-C.H.); 2 Fifth Branch office, Centers for Disease Control, Ministry of Health and Welfare, Kaohsiung 813, Taiwan; 3 Institute of Pharmacology, College of Medicine, Kaohsiung Medical University, Kaohsiung 807, Taiwan; E-Mail: chiu358@yahoo.com.tw; 4 Division of Environmental Health and Occupational Medicine, National Health Research Institute, Miaoli 350, Taiwan

**Keywords:** stroke, influenza, vaccination, hospitalization, revaccination, case-control

## Abstract

The aim of this study was to investigate the effect of influenza vaccination (and annual revaccination) on the risk of stroke admissions. We conducted a population-based case-control study in Taiwan. Cases consisted of patients >65 years of age who had a first-time diagnosis of stroke during the influenza seasons from 2006 to 2009. Controls were selected by matching age, sex, and index date to cases. Multiple logistic regression analysis was used to calculate the adjusted odds ratios (ORs) and 95% confidence intervals (CIs). Ever vaccinated individuals in the current vaccination season were associated with a reduced risk of ischemic stroke admissions (OR = 0.76, 95% CI = 0.60–0.97). Compared with individuals never vaccinated against influenza during the past 5 years, the adjusted ORs were 0.92 (95% CI = 0.68–1.23) for the group with 1 or 2 vaccinations, 0.73 (95% CI = 0.54–1.00) for the group with 3 or 4 vaccinations, and 0.56 (95% CI = 0.38–0.83) for the group with 5 vaccinations. There was a significant trend of decreasing risk of ischemic stroke admissions with an increasing number of vaccinations. This study provides evidence that vaccination against influenza may reduce the risk of hospitalization for ischemic stroke and that annual revaccination provides greater protection.

## 1. Introduction

Influenza can cause serious morbidity and mortality, especially among the elderly population (aged ≥ 65 years) [1,2]. Hospitalizations for stroke and cardiac diseases increase during influenza epidemics, but over half of the excess mortality during such epidemics was attributed to non-influenza causes, including cardiovascular diseases and stroke [3,4,5,6].

Influenza is an infectious disease that can induce inflammatory responses [7,8]. Evidence from basic and clinical research suggests that inflammatory mechanisms play a key role in the pathogenesis and progression of stroke [9]. Stroke is related with several risk factors, including influenza infection and acute and chronic respiratory tract infections [10,11]. Epidemiological studies have found a relationship between recent infection and acute stroke [12,13,14,15]. Many studies have shown that influenza vaccination can reduce hospitalizations for pneumonia and acute respiratory disease and deaths from all causes among the elderly [6,16,17,18,19,20]. However, only limited epidemiological studies have investigated the association between influenza vaccination and hospitalization for stroke among the elderly and the results have been inconsistent. Three previous studies found that influenza vaccination is associated with a significant reduction in the risk of hospitalization for stroke [4,18,21]. Two other studies, however, did not report a statistically association between influenza vaccination and risk of hospitalization for stroke [22,23].

Yearly influenza epidemics continue to be a major cause of annual substantial burden of illness including stroke in the elderly population [1]. Because epidemiological evidence for a link between influenza vaccination and risk of hospitalization for stroke is limited and no studies have addressed the benefit of annual revaccination against hospitalization for stroke, we undertook the present population-based case-control study in Taiwan to determine whether influenza vaccination is associated with a reduced risk of hospitalization for stroke among the elderly.

## 2. Experimental Section

### 2.1. Data Source

Taiwan’s National Health Insurance (NHI) program was implemented in Taiwan on 1 March 1995 and provides universal health coverage to all legal residents. Under the NHI, 98% of the island’s population is covered for a wide range of health care services including ambulatory care services, hospital admissions, traditional Chinese medicine, dental care, childbirth services, rehabilitation therapy, preventive services, home care, and care for chronic mental illness. From the population of all reimbursement claim records under the single payer system and using a systematic sampling method, the National Health Research Institute (NHRI) of Taiwan randomly sampled a representative panel database of 1,000,000 subjects in 2006 designed for research purposes. The NHRI reported no statistically significant differences in age, gender, and health care costs between the sample group and all enrollees [24]. This panel dataset (from January 1996 to December 2009) includes all claim data for these randomly drawn 1,000,000 subjects and enables us to explore the relationship between influenza vaccination and the risk of hospitalization for stroke. Previous epidemiological research has been conducted with this dataset, and information on prescription use, diagnoses, and hospitalizations has been confirmed to be of high reliability [25,26,27].

This study was exempt from full review by the Institution Review Board because all personally identifiable information has been removed by encrypting individuals' unique national identification numbers.

### 2.2. Identification of Cases and Controls

Cases consisted of all patients ≥65 years of age who were admitted to hospitals with a principal diagnosis of ischemic stroke (IS; International Classification of Diseases, 9th revision, Clinical Modification (ICD-9-CM) [28], Codes 433–435) or primary intracerebral hemorrhagic stroke (PIH; codes 431–432) during the influenza season of 2006 and 2009, and who had no previous diagnosis of stroke. Subjects who succumbed to stroke within two weeks from the date of vaccination were excluded because it will take about two weeks for immunization to be complete following influenza vaccination. According to the Taiwan Center for Disease Control, the influenza season in Taiwan (the peak of influenza virus activity) is generally between 1 December and 31 March in a typical year [19]. From the database, we randomly selected five population controls for each case. Each control was without a diagnosis of stroke at the date of the case’s hospitalization. Control patients were matched to the cases by sex, year of birth, and index date, and had no previous diagnosis of stroke. Population controls, selected using risk set sampling, (*i.e.*, to choose controls from the set of people in the source population who are at risk of becoming a case at the time at which the case is diagnosed), were assigned an index date identical to the index date (date of first-time diagnosis of stroke) of their matched case [29]. We also assessed the effect of influenza vaccination on the risk of stroke admissions during the summer season (June through September). This period was selected as a control period.

### 2.3. Exposure Definition

In Taiwan, influenza vaccination is free of charge for the entire elderly population (≥65 years of age) since October 2001. Information on all influenza vaccinations (code V04.8) was extracted from the NHRI database. The period of influenza vaccination season extended from 1 October through 31 December of each calendar year. Vaccinations during the winter months after 1 January were also acknowledged if they were performed before stroke events occurred.

### 2.4. Potential Confounders

We obtained, for all subjects in the study group, potential confounders that are documented risk factors for stroke, such as diabetes (code 250), hypertension (codes 401–405), hyperlipidemia (code 272), coronary heart disease (codes 410–414), atrial fibrillation (code 427.31), chronic rheumatic heart disease (codes 393–398), and other forms of heart disease (codes 420–429). All such confounders were recorded between 1 January 1996, and index date. Furthermore, the number of hospitalizations and the number of outpatient visits 1 year before the index date were treated as confounders.

### 2.5. Statistics

For comparisons of proportions, chi-square statistics were used. A multiple conditional logistic regression model was used to estimate the relative magnitude in relation to vaccination status adjusting for the aforementioned potential confounders. Odds ratios (ORs) and their 95% confidence intervals (CIs) were calculated using patients with no influenza vaccination as the reference. The number of vaccination within the previous 5 years was divided into four categories (0, 1–2; 3–4; 5). In the logistic regression model, we tested for linear trend by constructing this variable as a multinomial variable (with the value 0–3). All statistical analyses were performed using the SAS statistical software (Version 9.2, SAS Institute, Cary, NC, USA), and all statistical tests were two-sided. Statistical significance was determined at conventional values of *p* < 0.05.

## 3. Results and Discussion

Records of 520 stroke hospitalizations cases and 2,600 selected matched controls are included in the analyses. Table 1 presents the distribution of demographic characteristics and selected medical conditions of the stroke cases and controls. The mean age was 74.99 years for stroke patients and 74.98 for the controls. The case group had a significantly high rate of hypertension. However, cases and controls did not differ significantly with respect to diabetes, coronary heart disease, hyperlipidemia, atrial fibrillation, chronic rheumatic heart disease, and other forms of heart disease.

**Table 1:** Demographic characteristics of stroke cases and controls.

Variable	Cases (n = 520)	Controls (n = 2,600)	OR (95% CI)
Age (mean ± SD)	74.99 ± 6.77	74.98 ± 6.76	-
Female sex (%)	238 (45.77)	1,190 (45.77)	-
No. of hospitalizations	0.53 ± 1.03	0.28 ± 0.77	*p* < 0.001
No. of outpatient visits	28.61 ± 24.43	28.61 ± 22.78	*p* = 1.0
Diabetes (%)	214 (41.15)	987 (37.96)	1.14 (0.94–1.38)
Hypertension (%)	415 (79.81)	1,921 (73.88)	1.40 (1.11–1.76)
Hyperlipidemia	215 (41.35)	1,059 (40.73)	1.03 (0.75–1.24)
Coronary heart disease	255 (49.04)	1,240 (47.69)	1.06 (0.87–1.27)
Atrial fibrillation	53 (10.19)	245 (9.42)	1.09 (0.80–1.49)
Chronic rheumatic heart disease	20 (3.85)	128 (4.92)	0.77 (0.48–1.25)
Other forms of heart disease	229 (44.04)	1,058 (40.69)	1.15 (0.95–1.39)

The relationship between influenza vaccination and stroke admissions is shown in Table 2. Approximately 34.42% (179/520) of the cases and 40.58% (1,055/2,600) of the controls had been vaccinated against influenza during the current vaccination season. Ever vaccinated individuals in the current vaccination season were associated with a reduced risk of stroke admissions (PIH + IS) (OR = 0.76, 95% CI = 0.62–0.93). Adjustment for possible confounders (namely matching variables, hypertension, diabetes, hyperlipidemia, coronary heart disease, atrial fibrillation, chronic rheumatic heart disease, other forms of heart disease, number of hospitalizations and number of outpatient visits) only slightly altered the OR (the inverse association was somewhat weaker) (OR = 0.80, 95% CI = 0.64–0.98). When we analyzed the data separating PIH and IS, a significant inverse association was observed only for IS (OR = 0.76, 95% CI = 0.60–0.97). Influenza vaccination was not associated with a significant reduction in the risk of hospitalization for stroke (PIH + IS) during the summer months (OR = 0.89, 95% CI = 0.74–1.07).

**Table 2:** Associations of influenza vaccination with the risk of stroke hospitalization in a population-based case-control study, 2006–2009, Taiwan.

	No. of Cases/No. of Controls	Crude OR (95% CI)	Adjusted OR ^a^ (95% CI)
*Vaccinated during the current vaccination season*			
PIH			
No	67/320	1.00	1.00
Yes	43/230	0.88 (0.57–1.37)	0.89 (0.56–1.42)
IS			
No	274/1,225	1.00	1.00
Yes	136/825	0.73 (0.58–0.91)	0.76 (0.60–0.97)
PIH + IS			
No	341/1,545	1.00	1.00
Yes	179/1,055	0.76 (0.62–0.93)	0.80 (0.64–0.98)
***Number of vaccinations within the previous 5 years***			
PIH			
0	44/189	1.00	1.00
1–2	29/84	1.02 (0.58–1.79)	1.04 (0.57–1.88)
3–4	21/46	0.55 (0.30–1.01)	0.54 (0.28–1.03)
5	16/1	0.64 (0.33–1.27)	0.69 (0.34–1.40)
**X^2^ for trend = 3.04, *p* = 0.08**			
IS			
0	156/703	1.00	1.00
1–2	109/467	1.00 (0.76–1.34)	0.92 (0.68–1.23)
3–4	98/533	0.78 (0.58–1.05)	0.73 (0.54–1.00)
5	47/347	0.57 (0.40–0.83)	0.56 (0.38–0.83)
**X^2^ for trend = 9.97, *p* = 0.001**			
PIH + IS			
0	200/892	1.00	1.00
1–2	138/579	1.01 (0.78–1.30)	0.93 (0.72–1.22)
3–4	119/684	0.73 (0.56–0.95)	0.70 (0.53–0.92)
5	63/445	0.59 (0.43–0.82)	0.59 (0.42–0.83)
**X^2^ for trend = 17.61, *p* = 0.001**			

When exposure was categorized by cumulative number of influenza vaccinations within the previous 5 years, the adjusted ORs were 0.93 (95% CI = 0.72–1.22) for the group with one or two vaccinations, 0.70 (95% CI = 0.53–0.92) for the group with 3 or 4 vaccinations, and 0.59 (95% CI = 0.42–0.83) for the group with five vaccinations compared with never-vaccinated individuals. There was a significant trend of decreasing risk of hospitalization for stroke with increasing number of vaccinations (chi-square for linear trend = 12.61; *p* for trend < 0.001). The estimated ORs for IS were 0.92 (95% CI = 0.68–1.23), 0.73 (95% CI = 0.54–1.00), and 0.56 (95 CI = 0.38–0.83), respectively (chi-square for linear trend = 9.97; *p* for trend = 0.001). Again, the association for PIH was not significant.

In this population-based case-control study, we found that influenza vaccination in the current vaccination season was associated with a 24% reduction in the risk of IS. Vaccination every year for the previous 5 years was associated with a strong decrease in the risk of hospitalization for IS (OR = 0.56, 95% CI = 0.38–0.83). We also found that there was a significant trend of decrease in the risk of hospital admissions with increasing cumulative number of vaccinations after controlling for potential confounders.

Our findings are consistent with those of three studies that reported a significant decrease in the risk of hospitalization for stroke associated with influenza vaccination. In a case-control study conducted in France that consisted of 90 ischemic stroke patients and 180 controls ≥60 years, Lavallee *et al.* found that influenza vaccination during the current vaccination season was associated with a statistically significant reduction in the risk of stroke admissions by 50% (OR = 0.50, 95% CI = 0.26–0.94) [21]. In a large observational study conducted in the United States using ≥65-year-old members of three large managed-care organizations as study cohorts, Nichol *et al.* reported an odds ratio of 0.77 (95% CI = 0.66–0.89) for cerebrovascular disease in relation to influenza vaccination [18]. A German case-control study among 370 patients with ischemic or hemorrhagic stroke or transient ischemic stroke (TIA) and 370 community controls showed that vaccination against influenza during the recent influenza vaccination season was associated with a 54% reduction in risk of stroke (OR = 0.46, 95% CI = 0.28–0.77) [4].

It has been proposed that annual influenza revaccination may be a viable strategy to increase vaccination effectiveness [30,31]. However, the results have been inconsistent. Some studies reported clinical benefits (all-cause mortality) of annual revaccination [32,33,34] and others reported no additional protection against influenza infection [35,36,37]. To the best of our knowledge, this is the first study to report that more antecedent influenza vaccinations in the previous 5 years provide greater protection against hospitalization for stroke.

Prior studies have shown that recent or acute influenza infections were associated with an increased risk of stroke [12,13,14,15,38]. Stroke-related hospitalization could plausibly be reduced by preventing infections associated with influenza viruses and concomitant bacterial infections through influenza vaccination [4]. Research has not clearly identified the mechanism of the potential benefit from influenza vaccination, but such protection may reflect reduced immune activation of atherosclerotic plaque or coagulation [8,9,39]. Our results may contribute to the literature by providing indirect evidence that infections or inflammation may be causally related to stroke [21].

Investigations have shown that influenza vaccinations are protective against stroke admissions [4,18,21]. Some studies have suggested a link between influenza and stroke-related events [10,11,40]. However, this relationship has not been well characterized. A recent study at the population level conducted by Foster *et al.* reported a lack of association between influenza and strokes [41]. It must be noted that the above mentioned study was an ecological study. In this study, the authors analyzed the correlation by using the aggregate incidence for stroke rather than associations at the individual level. Using aggregate incidence may confound their results, as it is possible that the positive findings between stroke and influenza vaccination study my instead be due to unobservable differences or other types of selection bias between subjects who chose or declined annual vaccination.

A particular strength of our study is the use of a randomly selected sample that is highly representative of the population in Taiwan. We are able to eliminate selection bias in our study because we included all patients newly diagnosed with stroke from 2006 to 2009, and because we selected the control subjects from a simple random sampling of the general population of insured individuals in Taiwan. Because vaccination data were obtained from an historical database that collects all medical information before the date of stroke admissions, recall bias for the vaccination status and the number of vaccinations was avoided.

However, several limitations of the present study should be noted. First, a number of potential confounding factors which may be associated with stroke, such as smoking, alcohol use, diet, body mass index, and family history of stroke, were not included in our database because each variable are unavailable in a medical claims database designed primarily for processing reimbursement; Second, stroke diagnoses that rely on administrative claims data reported by physicians or hospitals may be less accurate than diagnoses made according to standardized criteria. However, the validity of stroke diagnoses is considered excellent because computed tomography (CT) and/or magnetic resonance imaging (MRI) are performed in almost all stroke cases in Taiwan [42]; Third, information bias may have occurred if the vaccination was not recorded. However, such misclassification would likely be random because vaccination data is recorded before stroke admissions. Any such misclassification was likely non-differential, which would underestimate rather than overestimate our study findings; Fourth, the patients in our database are mainly Han Chinese ethnicity. This fact may somewhat restrict the generalizability of these findings to other locations with different ethnic composition because ethnicity-based differences in the pattern of stroke have been observed [43]. In addition, because the study population included only elderly persons, the results may not be generalized to persons <65 years of age; Fifth, due to data limitations, there was no information on the socioeconomic status of our study subjects. However, concerns over confounding by socioeconomic status should be minimal in Taiwan because the NHI system provides comprehensive coverage to all residents regardless of ability to pay, and gives patients access to any clinic or hospital freely even without a referral from a general practitioner. People in Taiwan have nearly no barriers to medical service in terms of accessibility and costs [44]. However, many people did not receive vaccination. The respective rates of vaccination against influenza were 34.42% for the cases and 40.58% for the controls during the current vaccination season. The reasons are not clear. Selection bias might have occurred because the elderly who were extremely frail or very healthy might not have received vaccinations. In addition, unobservable confounding in personal health management choices and the decision to vaccinate remains likely because the vaccination was still voluntary; Sixth, it may be argued that vaccinated subjects on the one hand are the persons who are likely to have better lifestyle habits and are most concerned about health problems and who might have a worse state of health [21]. The confounding effect of medical attention could be controlled for in the conditional logistic regression model by using, as proxies for the propensity to engage in personal health management, the number of hospitalizations and number of physician visits. In addition, awareness of the benefits of influenza vaccination may have an important effect on influenza vaccination. However, there is no reason to believe that this would be different for cases and controls because the possible role of influenza vaccination as a protective factor for stroke has not received public attention; Seventh, our measures of the healthy-user effect were fairly rudimentary and limited. It would have been ideal had we also had access to more detailed information about subjects’ health screening and health-promoting habits, including adherence to medications. It has been hypothesized that influenza vaccination could improve outcomes either by strengthing innate immunity [45] or by virtue of the fact that vaccination prevents influenza infection and leads to a shift in the microbial spectrum toward far less virulent pathogens [46]. We believe, despite speculations, that our results are broadly consistent with difficult-to-control confounding [47]; Lastly, as with any observational study, omitted variable bias is also possible as we are unable to control for difference between cases and controls.

## 4. Conclusions

In summary, our study results provides evidence suggesting that vaccination against influenza may lower the risk of hospitalization for IS, and that annual revaccination may confer greater protection against IS-associated hospitalization. This study supports strategies to increase the vaccination rates and to assure yearly influenza revaccination for the elderly as a potential measure to reduce hospitalizations for IS.
